# Triterpenoids From *Alisma* Species: Phytochemistry, Structure Modification, and Bioactivities

**DOI:** 10.3389/fchem.2020.00363

**Published:** 2020-04-30

**Authors:** Pengli Wang, Tongxin Song, Rui Shi, Mingshuai He, Rongrong Wang, Jialin Lv, Miaomiao Jiang

**Affiliations:** Tianjin Key Laboratory of TCM Chemistry and Analysis, Institute of Traditional Chinese Medicine, Tianjin University of Traditional Chinese Medicine, Tianjin, China

**Keywords:** triterpenoids, *Alisma*, structure, anticancer, lipid-regulation

## Abstract

Plants from *Alisma* species belong to the genus of *Alisma* Linn. in *Alismataceae* family. The tubers of *A. orientale* (Sam.) Juzep, also known as *Ze Xie* in Chinese and *Takusha* in Japanese, have been used in traditional medicine for a long history. Triterpenoids are the main secondary metabolites isolated from *Alisma* species, and reported with various bioactive properties, including anticancer, lipid-regulating, anti-inflammatory, antibacterial, antiviral and diuretic activities. In this brief review, we aimed to summarize the phytochemical and pharmacological characteristics of triterpenoids found in *Alisma*, and discuss their structure modification to enhance cytotoxicity as well.

## Introduction

Plants from the genus of *Alisma* Linn. (*Alismataceae*) are widely distributed in temperate regions and subtropics of the northern hemisphere, belonging to 11 species. Six species were found in China and Asia, including *A. canaliculatum, A. gramineum, A. nanum, A. orientale, A. plantago-aquatica* and *A. lanceolatum* (Flora of China Committee, 1992). The tubers of *A. orientale*, known as *Ze Xie* in Chinese or *Takusha* in Japanese, have been used as diuretic and detumescent medications for a long history (Chinese Pharmacopoeia Commission, 2015). It is also used to treat obesity, diabetes and hyperlipidemia nowadays.

Phytochemical studies have revealed that triterpenoids are dominant components in tubers of *Alisma* plants. A total of 118 triterpenoids have been isolated and identified from *Alisma* species so far. Most of them contain protostane tetracyclic aglycones, whereas glycosides are rarely found in other plants. These triterpenoids have been considered as chemotaxonomic markers of the genus (Zhao et al., 2007). A small amount of other kinds of compounds have also been isolated from A. orientale, including diterpenoids, sesquiterpenoids, polysaccharides, phytosterols, amino acids, flavonoids and fatty acids (Zhang et al., 2017). The presence of triterpenoids attributes to the bioactivities of A. orientales (Tian et al., 2014; Shu et al., 2016), such as alisol A 24-acatate (**2**), and alisol B 23-acetate (**47**) (Choi et al., 2019).

Alisols have shown a series of biological activities, such as anticancer (Law et al., 2010), lipid-regulating (Cang et al., 2017), anti-inflammatory (Kim et al., 2016), antibacterial (Jin et al., 2012), antiviral (Jiang et al., 2006), and diuretic activities (Zhang et al., 2017). Since alisol B 23-acetate (**47**) exhibits a significant anti-tumor activity, structure-based modification on alisol B 23-acetate (**47**) gives a profound change of activity.

This paper aims to systematically review triterpenoids from *Alisma* species, involving their phytochemical characteristics, biosynthesis, bioactivities and structure modification.

## Triterpenoids

Starting from 1968, triterpenoids have been isolated from *Alisma* genus successively (Murata et al., 1968). All these compounds contain protostane tetracyclic skeleton with the structural characteristics of *trans*-fusions for A/B, B/C and C/D rings, α-methyl submitted at C-8, β-methyl at C-10, β-methyl at C-14 and side chain at C-17. At present there are 101 protostane triterpenoids, 12 nor-protostanes, and 5 seco-protostanes reported from *Alisma*. According to the changes of side chains submitted at C-17, protostane triterpenoids from *Alisma* are divided into four classes, including open aliphatic chains, epoxy aliphatic chains, spiro hydrocarbon at C-17, and epoxy at C-16, C-23 or C-16, C-24. The individual triterpenoids were detailed in Table 1.

**Table 1:** A total of 118 triterpenoids isolated and identified from *Alisma* genus.

**No**.	**Name**	**Skeleton structure**	**R_**1**_**	**R_**2**_**	**R_**3**_**	**R_**4**_**	**R_**5**_**	**R_**6**_**	**Double bond position**	**Source**	**References**
**PROTOSTANES WITH OPEN ALIPHATIC CHAINS AT C-17**											
1	alisol A	A	βOH	H	βOH	βOH	OH	H	Δ^13(17)^	*A. orientalis*	Peng et al., 2002a
2	alisol A 24-acetate	A	βOH	H	βOH	βOAc	OH	H	Δ^13(17)^	*A. orientalis*	Peng et al., 2002a
3	alisol A 23-acetate	A	βOH	H	βOAc	βOH	OH	H	Δ^13(17)^	*A. orientalis*	Peng et al., 2002a
4	11-deoxyalisol A	A	H	H	βOH	βOH	OH	H	Δ^13(17)^	*A. orientalis*	Peng et al., 2002b
5	23-o-methyl alisol A	A	βOH	H	βOMe	βOH	OH	H	Δ^13(17)^	*A. orientale*	Nakajma et al., 1994
6	25-o-methoxy-alisol A	A	βOH	H	βOH	βOH	OMe	H	Δ^13(17)^	*A. orientale*	Nakajma et al., 1994
7	16-oxo-alisol A	A	βOH	O	βOH	βOH	OH	H	Δ^13(17)^	*A. orientale*	Mai et al., 2015
8	16-oxo-alisol A-23-acetate	A	βOH	O	βOAc	βOH	OH	H	Δ^13(17)^	*A. orientale*	Zhao et al., 2015
9	16-oxo-alisol A-24-acetate	A	βOH	O	βOH	βOAc	OH	H	Δ^13(17)^	*A. orientale*	Zhao et al., 2015
10	16-oxo-11-deoxy- alisol A	A	H	O	βOH	βOH	OH	H	Δ^13(17)^	*A. orientale*	Mai et al., 2015
11	5β,29-dihydroxy alisol A	A (5βOH)	βOH	H	βOH	βOH	OH	OH	Δ^13(17)^	*A. plantago-aquatica*	Wang et al., 2017b
12	25-o-butyl alisol A	A	βOH	H	βOH	βOH	OBu	H	Δ^13(17)^	*A. orientalis*	Zhang et al., 2017
13	alisol E	A	βOH	H	βOH	αOH	OH	H	Δ^13(17)^	*A. orientale*	Yoshikawa et al., 1993
14	alisol E-23-acetate	A	βOH	H	βOAc	αOH	OH	H	Δ^13(17)^	*A. orientale*	Yoshikawa et al., 1993
15	alisol E-24-acetate	A	βOH	H	βOH	αOAc	OH	H	Δ^13(17)^	*A. orientale*	Yoshikawa et al., 1993
16	25-o-ethylalisol A	A	βOH	H	βOH	βOH	OEt	H	Δ^13(17)^	*A. orientale*	Mai et al., 2015
17	alisol H	A	H	O	O	H	OH	H	Δ^13(17)^	*A. orientale*	Yoshikawa et al., 1999
18	16β-methoxyalisol E	A	βOH	βOMe	βOH	αOH	OH	H	Δ^13(17)^	*A. orientale*	Li et al., 2017
19	16β,25-dimethoxyalisol E	A	βOH	βOMe	βOH	αOH	OMe	H	Δ^13(17)^	*A. orientale*	Li et al., 2017
20	16β-hydroperoxyalisol E	A	βOH	βOOH	βOH	αOH	OH	H	Δ^13(17)^	*A. orientale*	Li et al., 2017
21	11,24-dihydroxy-alisol H	A	βOH	O	O	βOH	OH	H	Δ^13(17)^	*A. orientale*	Yoshikawa et al., 1999
22	alisol T	A	βOH	βOMe	OH	H	OH	H	Δ^13(17)^	*A. orientale*	Li et al., 2017
23	alismanin I	A	βOH	H	O	OH	H	H	Δ^13(17)^	*A. orientale*	Yi et al., 2019
24	15,16-dihydroalisol A.	A	βOH	H	βOH	βOH	OH	H	Δ^13(17), 15(16)^	*A. orientale*	Mai et al., 2015
25	alismanol D	A	H	H	H	αOH	OH	H	Δ^9(11), 12(13)^	*A. orientale*	Mai et al., 2015
26	24-epi-alismanol D	A	H	H	H	βOH	OH	H	Δ^9(11), 12(13)^	*A. orientalis*	Xin et al., 2018
27	alismanol A	A	H	O	O	αOH	OH	H	Δ^11(12), 13(17)^	*A. orientale*	Mai et al., 2015
28	alismanol C	A	H	O	βOAc	αOH	OH	H	Δ^11(12), 13(17)^	*A. orientale*	Mai et al., 2015
29	16-oxo-11-anhydro alisol A	A	H	O	βOH	βOH	OH	H	Δ^11(12), 13(17)^	*A. orientale*	Mai et al., 2015
30	16-oxo-11-anhydroalisol A 24-acetate	A	H	O	βOH	βOAc	OH	H	Δ^11(12), 13(17)^	*A. orientale*	Ma et al., 2016
31	3-oxo-11β,23-dihydroxy-24,24-dimethyl−26,27-dinorprotost-13(17)-en-25-oic-acid	A	βOH	O	H	βOH	COOH	H	Δ^13(17)^	*A. orientale*	Zhao et al., 2013
32	alismanin B	A	βOH	O	H	βOH	H	H	Δ^13(17)^	*A. orientale*	Wang et al., 2017a
33	25-anhydroalisol A	B	βOH	H	βOH	βOH			Δ^13(17)^	*A. orientalis*	Peng et al., 2002a
34	11-acetate-25-anhydroalisol A	B	βOAc	H	βOH	βOH			Δ^13(17)^	*A. orientalis*	Peng et al., 2002a
35	24-acetate-25-anhydroalisol A	B	βOH	H	βOH	βOAc			Δ^13(17)^	*A. orientalis*	Peng et al., 2002a
36	11-deoxy-25-anhydro-alisol E.	B	H	H	βOH	αOH			Δ^13(17)^	*A. orientale*	Mai et al., 2015
37	alisol X	B	βOH	H	H	O			Δ^13(17)^	*A. orientale*	Xu et al., 2012
38	23-acetate-25-anhydroalisol E	B	H	H	βOAc	αOH			Δ^13(17)^	*A. orientalis*	Han et al., 2013
39	24-acetate-25-anhydroalisol E	B	H	H	βOH	αOAc			Δ^13(17)^	*A. orientalis*	Han et al., 2013
40	alismanol B	B	H	O	βOH	αOH			Δ^11(12), 13(17)^	*A. orientale*	Mai et al., 2015
41	7-oxo-16-oxo-11-anhydro alisol A	C								*A. orientale*	Mai et al., 2015
42	alismanol M	D								*A. orientale*	Xin et al., 2016
43	13,17-epo-alisol A	E	βOH	αOH						*A. orientalis*	Peng et al., 2002b
44	13,17-epoalisol A 24-acetate	E	βOH	αOAc						*A. orientalis*	Peng et al., 2002b
45	11-deoxy-13,17-epoxy-alisol A	E	H	βOH						*A. orientale*	Nakajma et al., 1994
**PROTOSTANES WITH EPOXY ALIPHATIC CHAINS AT C-17**											
46	alisol B	F	βOH	H	H	H	αMe	βOH	Δ^13(17)^	*A. orientale*	Nakajma et al., 1994
47	alisol B 23-acetate	F	βOH	H	H	H	αMe	βOAc	Δ^13(17)^	*A. orientale*	Nakajma et al., 1994
48	11-deoxy-alisol B-23-acetate	F	H	H	H	H	βMe	βOAc	Δ^13(17)^	*A. orientale*	Nakajma et al., 1994
49	11-deoxy-alisol B	F	H	H	H	H	βMe	βOH	Δ^13(17)^	*A. orientale*	Nakajma et al., 1994
50	16β-acetoxy alisol B	F	βOH	H	βOAc	H	αMe	βOH	Δ^13(17)^	*A. orientalis*	Cang et al., 2017
51	16α-acetoxy alisol B	F	βOH	H	αOAc	H	αMe	βOH	Δ^13(17)^	*A. orientalis*	Cang et al., 2017
52	16β-hydroxyalisol B-23-acetate	F	βOH	H	βOH	H	αMe	βOAc	Δ^13(17)^	*A. orientalis*	Peng and Lou, 2001
53	16β-methoxyalisol B-23- acetate	F	βOH	H	βOMe	H	αMe	βOAc	Δ^13(17)^	*A. orientale*	Jin et al., 2012
54	16β-ethoxy alisol B 23-acetate	F	βOH	H	βOEt	H	αMe	βOAc	Δ^13(17)^	*A. orientalis*	Zhang et al., 2017
55	alisol C	F	βOH	H	O	H	αMe	βOH	Δ^13(17)^	*A. orientale*	Nakajma et al., 1994
56	11-deoxy-alisol C-23-acetate	F	H	H	O	H	αMe	βOAc	Δ^13(17)^	*A. orientale*	Nakajma et al., 1994
57	11-deoxy-alisol C	F	H	H	O	H	αMe	βOH	Δ^13(17)^	*A. plantago-aquatica*	Fukuyama et al., 1988
58	20-hydroxyalisol C	F	βOH	H	O	OH	αMe	βOH	Δ^13(17)^	*A. orientale*	Mai et al., 2015
59	alisol C 23-acetate	F	βOH	H	O	H	αMe	βOAc	Δ^13(17)^	*A. plantago-aquatica*	Fukuyama et al., 1988
60	alisol M-23-acetate	F	βOH	βOH	O	H	αMe	βOAc	Δ^13(17)^	*A. orientale*	Li et al., 2017
61	alisol N-23-acetate	F	βOH	βOH	H	H	αMe	βOAc	Δ^13(17)^	*A. orientale*	Li et al., 2017
62	16β-hydroperoxyalisol B	F	βOH	H	βOOH	H	αMe	βOH	Δ^13(17)^	*A. orientale*	Li et al., 2017
63	16β-hydroperoxyalisol B 23-acetate	F	βOH	H	βOOH	H	αMe	βOAc	Δ^13(17)^	*A. orientale*	Li et al., 2017
64	alisol L	F	H	H	O	H	αMe	βOH	Δ^11(12), 13(17)^	*A. orientale*	Zhao et al., 2015
65	alisol L-23-acetate	F	H	H	O	H	αMe	βOAc	Δ^11(12), 13(17)^	*A. orientale*	Yoshikawa et al., 1999
66	13β,17β-epoxy-alisol B	G	βOH	βOH						*A. orientale*	Nakajma et al., 1994
67	13β,17β-epoxy-23- acetate-alisol B	G	βOH	βOAc						*A. orientale*	Jin et al., 2012
68	11-deoxy-13β,17β-epoxy-alisol B 23-acetate	G	H	βOAc						*A. orientale*	Nakajma et al., 1994
69	alisol D	G	βOH	αOAc						*A. plantago-aquatica*	Fukuyama et al., 1988
70	alisol D 11-acetate	G	βOAc	αOAc						*A. plantago-aquatica*	Fukuyama et al., 1988
71	11-deoxyalisol D	G	H	αOAc						*A. orientale*	Yoshikawa et al., 1999
72	alisol J−23 acetate	H								*A. orientale*	Yoshikawa et al., 1999
73	alisol K-23-acetate	I								*A. orientale*	Yoshikawa et al., 1999
74	alismanol O	J	H							*A. orientale*	Xin et al., 2016
75	alismanol P	J	αOH							*A. orientale*	Xin et al., 2016
76	alisolide H	K								*A. plantago-aquatica*	Jin et al., 2019
77	alisolide G	L	O	αOAc						*A. plantago-aquatica*	Jin et al., 2019
78	alisol Q 23-acetate	L	O	βOAc						*A. orientale*	Jin et al., 2012
79	alisol S 23-acetate	L	βOH	βOAc						*A. orientale*	Li et al., 2017
80	alisolide I	M								*A. plantago-aquatica*	Jin et al., 2019
81	alismaketone A-23-acetate	N								*A. orientale*	Yoshikawa et al., 1997
**PROTOSTANES WITH SPIRO HYDROCARBON AT C-17**											
82	alismanol Q	O								*A. orientale*	Xin et al., 2016
83	alisol U	P								*A. orientale*	Li et al., 2017
84	alisol V	Q								*A. orientale*	Li et al., 2017
85	alisolide D	R								*A. plantago-aquatica*	Jin et al., 2019
86	alisolide E	S	βOH						Δ^12(13)^	*A. plantago-aquatica*	Jin et al., 2019
87	alisolide F	S	H						Δ^9(11), 12(13)^	*A. plantago-aquatica*	Jin et al., 2019
88	neoalisol	T	βOH	βOH						*A. orientalis*	Peng et al., 2002a
89	neoalisol 11.24-diacetate	T	βOAc	βOAc						*A. orientalis*	Peng et al., 2002a
**PROTOSTANES WITH FUSED RING AT C-16 AND C-17**											
90	16,23-oxidoalisol B	U	βOH						Δ^13(17)^	*A. orientale*	Nakajma et al., 1994
91	alisol I	U	βH						Δ^13(17)^	*A. orientale*	Yoshikawa et al., 1999
92	alisol F	V	βOH	βOH	OH				Δ^13(17)^	*A. orientale*	Yoshikawa et al., 1993
93	alisol F-24-acetate	V	βOH	βOAc	OH				Δ^13(17)^	*A. orientalis*	Peng and Lou, 2001
94	25-o-methylalisol F	V	βOH	βOH	OMe				Δ^13(17)^	*A. orientalis*	Chen et al., 2018
95	11-anhydroalisol F	V	H	βOH	OH				Δ^11(12), 13(17)^	*A. orientalis*	Hu et al., 2008a
96	alisol O	V	H	βOAc	OH				Δ^11(12), 13(17)^	*A. plantago-aquatica*	Jiang et al., 2006
97	25-anhydroalisol F	W	βOH						Δ^13(17)^	*A. orientalis*	Hu et al., 2008a
98	11,25-anhydro-alisol F	W	H						Δ^11(12), 13(17)^	*A. orientalis*	Hu et al., 2008b
99	alismaketone B-23-acetate	X	βOH	αOAc					Δ^13(17)^	*A. orientale*	Matsuda et al., 1999
100	alismanol E	X	H	O					Δ^11(12), 13(17)^	*A. orientale*	Mai et al., 2015
101	alismanol J	Y								*A. orientalis*	Zhang et al., 2017
**NOR-PROTOSTANES**											
102	alismanol H	Z	H	Me						*A. orientalis*	Zhang et al., 2017
103	alismanin A	Z	C_6_H_5_	H						*A. orientale*	Wang et al., 2017a
104	alisolide A	a	O	βOH					C-17R	*A. plantago-aquatica*	Jin et al., 2019
105	alisolide B	a	O	βOOH					C-17S	*A. plantago-aquatica*	Jin et al., 2019
106	alisolide C	a	βOH	βOH					C-17S	*A. plantago-aquatica*	Jin et al., 2019
107	alisolide	b								*A. orientalis*	Xin et al., 2018
108	17-epi-alisolide	c								*A. orientalis*	Xin et al., 2018
109	alismanol F	d								*A. orientale*	Mai et al., 2015
110	alismanol G	e	H	O	Ac				Δ^11(12), 13(17)^	*A. orientale*	Mai et al., 2015
111	alismanol I	e	βOH	O	OH				Δ^13(17)^	*A. orientalis*	Zhang et al., 2017
112	alisol R	e	βOH	H	O				Δ^12(13)^	*A. orientale*	Li et al., 2017
113	13β,17β-epoxy-24,25,26,27 -tetranor-alisol A 23-oic acid	f								*A. orientale*	Zhao et al., 2007
**SECO-PROTOSTANES**											
114	alismanin C	g								*A. orientale*	Wang et al., 2017a
115	alismaketone C-23-acetate	h								*A. orientale*	Matsuda et al., 1999
116	alismalactone-23-acetate	i	H							*A. orientale*	Yoshikawa et al., 1997
117	3-methyl-alismalactone 23-acetate	i	Me							*A. orientale*	Yoshikawa et al., 1997
118	alisol P	j								*A. orientale*	Zhao et al., 2007

### Protostanes With Open Aliphatic Chains at C-17

**Figure 1:**
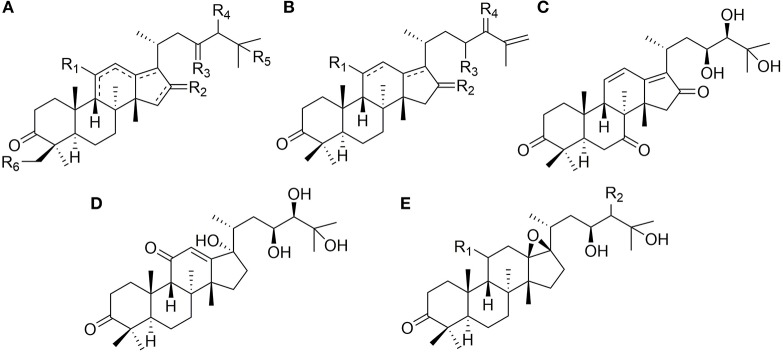
Chemical structures of the protostanes with open aliphatic chains at C-17.

Forty-five protostanes with open aliphatic chains at C-17 (**1**–**45**) have been identified as shown in Figure 1. Alisol A (**1**) is a representative compound of this type. Hydroxyl groups may substitute at C-29 (**11**) (Wang et al., 2017b), disubstitute at C-23/C-24 (**19**) and C-23/C-25 (**43-45**) (Nakajma et al., 1994; Peng et al., 2002b), or trisubstitute at C-23, C-24, and C-25 (**41**, **42**). The hydroxyl group at C-23 or C-24 is easily acetylated. Moreover, double bond may form at C-25 and C-26_**(38**, **39**) (Han et al., 2013), or C-25 may be substituted by carboxyl group (**31**) (Zhao et al., 2013).

Carbonyl groups substitute at C-16 (**8**, **9**) (Zhao et al., 2015), disubstitute at C-7/C-16 (**41**) (Mai et al., 2015) or C-16/C-23 (**21**) (Yoshikawa et al., 1999), or substitute at C-24 (**37**) (Xu et al., 2012) or C-23 (**23**) (Yi et al., 2019). Hydroxymethyl groups substitute at C-16 (**18**) (Li et al., 2017) or disubstitute at C-16/C-25 (**19**).

### Protostanes With Epoxy Aliphatic Chains at C-17

**Figure 2:**
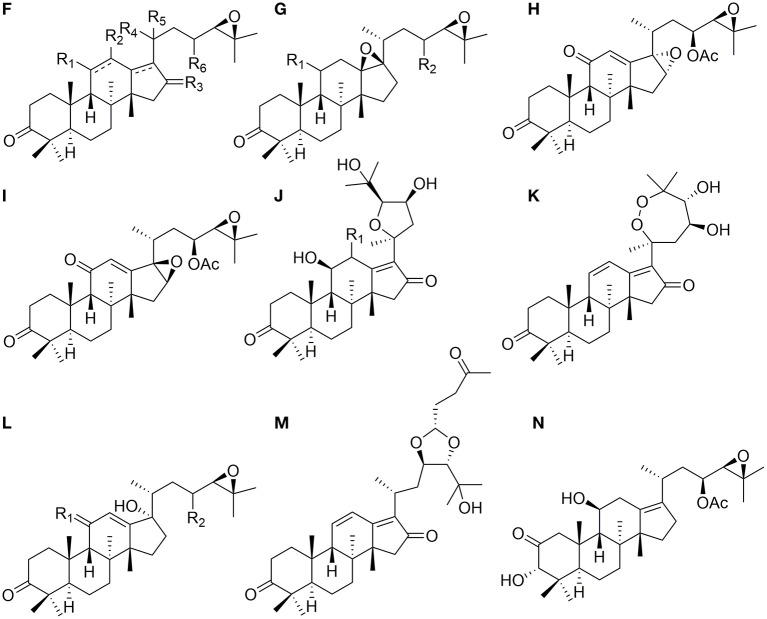
Chemical structures of the protostanes with epoxy aliphatic chains at C-17.

Thirty-six protostanes with epoxy aliphatic chains at C-17 (**46**–**81**) have been found in the genus of *Alisma* and their structures are listed in Figure 2. Alisol B 23-acetate (**47**) is a representative compound of this type. Epoxy group usually forms at C-24 and C-25 (**46–73**, **77–79**, **81**) (Fukuyama et al., 1988), and C-23 may be substituted by hydroxyl (**66**) or acetoxyl group (**67–71**).

Except for epoxy ring, tetrahydrofuran ring from C-20 to C-24 (**74**, **75**) and seven-membered peroxic ring from C-20 to C-25 (**76**) are also existed in the side chains at C-17.

### Protostanes With Spiro Hydrocarbon at C-17

**Figure 3:**
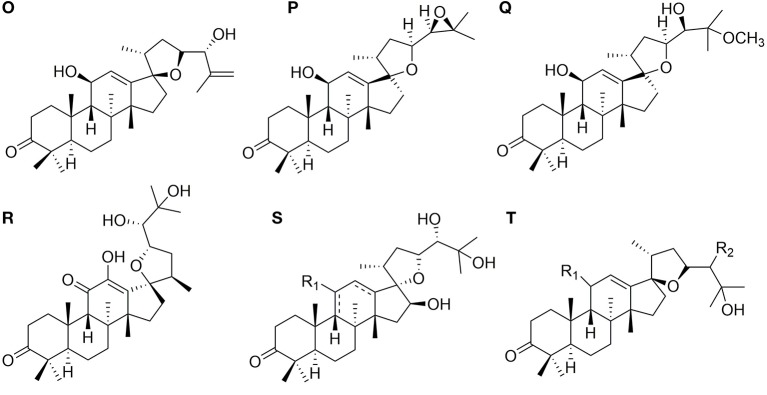
Chemical structures of the protostanes with spiro hydrocarbon at C-17.

Eight protostanes with spiro hydrocarbon at C-17 (**82–89**) have been isolated from the genus of *Alisma* as shown in Figure 3. Oxaspiro-nonane moiety is generated between D ring and its side chain with C-17 as spiro hydrocarbon. Methyl group substituted at C-20 with α- (**82**) (Xin et al., 2016) or β- (**85**) (Jin et al., 2019) conformation. Alisol U (**83**) differs from alisol V (**84**) by forming an epoxy at C-24 and C-25.

### Protostanes With Fused Ring at C-16 and C-17

**Figure 4:**
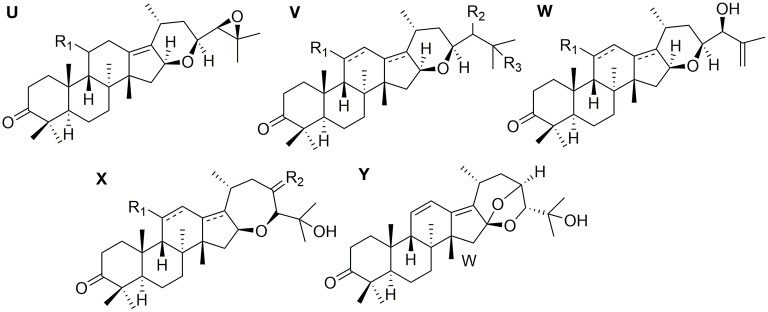
Chemical structures of the protostanes with fused ring at C-16 and C-17.

Twelve protostanes with fused-ring at C-16 and C-17 (**90**–**101**) have been isolated from *Alisma* as shown in Figure 4. Tetrahydropyrane ring is fused at C-16 and C-17 (**90–98**) (Yoshikawa et al., 1993; Peng and Lou, 2001; Hu et al., 2008a,b; Chen et al., 2018). Oxacycloheptane ring is fused at C-16 and C-17 (**99–101**). Alismanol J (**101**) differs from alismaketone B-23-acetate (**99**) by forming an oxygen bridge between C-16 and C-23.

### Nor- and seco-protostanes

Twelve nor-protostanes (**102**–**113**) have been found in *Alisma*, including two demethyl-protostanes (**102**, **103**) and ten tetranorprotostanes (**104**–**113**). Among C-2 may be submitted by carbanyl group (**109**) (Mai et al., 2015). The configuration of C-17 is determined (**107**, **108**) (Xin et al., 2018).

**Figure 5:**
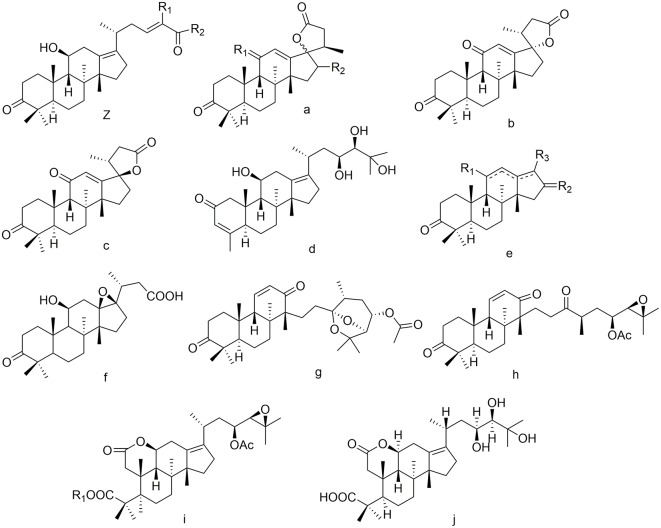
Chemical structures of the nor- and seco-protostanes.

Only five seco-protostanes (**114–118**) have been known in *Alisma*, including two 13, 17-seco-protostanes (**114**, **115**) (Matsuda et al., 1999; Wang et al., 2017a) and three 2, 3-seco-protostanes (**116–118**) (Yoshikawa et al., 1997). Their structures were detailed in Figure 5.

## Biosynthesis

**Figure 6:**
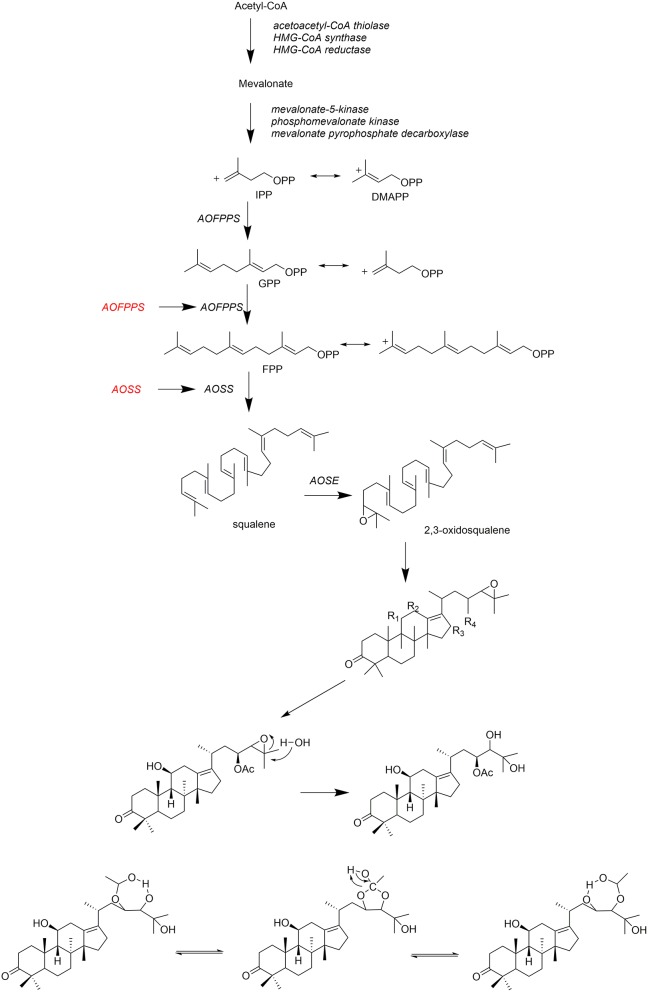
Biosynthesis pathway of *Alisma* triterpenoids.

*Alisma* triterpenoids is commonly biosynthesized through mevalonic aid (MVA) pathway (Zhang et al., 2018) as shown in Figure 6. Three molecules of acetyl-CoA are catalyzed by enzymes to form mevalonate acid (MVA) (Vinokur et al., 2014). It is catalyzed by mevalonate pyrophosphate decarboxylase to produce isopentyl pyrophosphate (IPP), which reacts with dimethylallyl pyrophosphate (DMAPP) to generate geranyl pyrophosphate (GPP) by farnesyl pyrophosphate synthase of A. orientale (AOFPPS) (Peng et al., 2018). Squalene is synthesized by squalene synyase of *A. orientale* (AOSS) (Shen et al., 2013), which is then catalyzed by squalene epoxidase of *A. orientale* (AOSE) to produce 2,3-oxidosqualeneand further to form protostane tetracyclic skeleton (Zhang et al., 2018). AOFPPS and AOSS are rate-limiting enzymes in *Alism*a triterpenoids biosynthesis pathway (Zhou et al., 2018).

Fresh materials of *A. orientalis* are naturally rich in alisol B 23-acetate (**47**) (Zhu and Peng, 2006), which can convert into alisol A 24-acetate (**2**), alisol A (**1**), and alisol B **(46)** after processing at high temperature (Zheng et al., 2006). Other triterpenoids, such as alisol A (**1**) (Peng et al., 2002a) and their derivatives, were formed during the drying process (Yoshikawa et al., 1994).

## Bioactivities

*Alisma orientale* is traditionally used to treat oliguria, edema, gonorrhea with turbid urine, leukorrhea, diarrhea, dizziness and hyperlipidemia (Chinese Pharmacopoeia Commission, 2015). Modern pharmacological studies have demonstrated its diuretic and lipid-lowering efficiency, together with anticancer, lipid-regulating, anti-inflammatory, antibacterial, antiviral activities.

### Anticancer Activities

Recently, the experiments *in vitro* highlight that alisols induce apoptosis and autophagy in human tumor cells, such as lung cancer (Wang et al., 2018), ovarian cancer (Zhang et al., 2016), and prostate cancer (Huang et al., 2006) cell lines. The cytotoxicities of alisol B 23-acetate (**47**), cancer cell lines, including L1210 and K562 leukemia alisol C 23-acetate (**59**), alisol B (**46**) and alisol A 24-acetate (**2**) are examined on several cells, B16-F10 melnoma cells, A549 lung adenocarcinoma cells, SK-OV3 ovarian cells, HT 1080 fibrosarcoma cells. The results show that alisol B 23-acetate (**47**), alisol C 23-acetate (**59**) and alisol A 24-acetate (**2**) have weaker inhibitory activities against all the tested cancer cells with ED_50_ values in the range of 10~20 μg/ml, while alisol B (**46**) exhibits significant effect on SK-OV3, B16-F10, and HT1080 with ED_50_ values of 7.5, 7.5, and 4.9 μg/ml, respectively (Lee et al., 2001). Moreover, alisol F 24-acetate (**93**) and alisol B 23-acetate (**47**) are found inducing cell apoptosis via inhibiting P-glycoprotein mediation and reversing the multidrug resistance in cancer cell lines (Wang et al., 2004; Hyuga et al., 2012; Pan et al., 2016).

Alisol B (**46**) targets on Ca^2+^-ATP enzymes in the sarcoplasmic reticulum or endoplasmic reticulum to induce autophagy of cancer cells (Law et al., 2010). This compound can also induce cell apoptosis by inhibiting the invasion and metastasis of SGC7901 cells (Xu et al., 2009).

Alisol B 23-acetate (**47**) can inhibit the proliferation of PC-3 prostate cancer (Huang et al., 2006), and induce the apoptosis of lung cancer A549 and NCI-H292 cells through the mitochondrial caspase pathway (Wang et al., 2018). Alisol B 23-acetate (**47**) obviously inhibits the proliferation, migration and invasion of ovarian cancer cell lines and induces accumulation of the G1 phase in a concentration-dependent manner. The protein levels of cleaved poly ADP-ribose polymerase (PARP) and the ratio of Bax/Bcl-2 are up-regulated, while the levels of CDK4, CDK6 and cyclin D1 are down-regulated after alisol B 23-acetate (**47**) treatment. Moreover, it can up-regulate the expression levels of IRE1α and Bip, and down regulate MMP-2 and MMP-9 in a dose- and time- dependent manner (Zhang et al., 2016). However, current studies of *Alisma* triterpenoids are limited into drug screening *in vitro*, and their anticancer activities need to be validated *in vivo*.

### Lipid-Lowering Effects

One of *A. orientale* traditional effects is to treat hyperlipidemia. Studies have shown that the extracts of A. orientale tubers have potential effects on hyperlipidemia diseases (Park et al., 2014; Jang et al., 2015; Li et al., 2016; Miao et al., 2017). Alisol B 23-acetate (**47**) and alisol A 24-acetate (**2**) reduce the levels of TC and LDL-C in hyperlipidemia mice via inhibiting the activity of HMG-CoA reductase (Murata et al., 1970; Xu et al., 2016). According to the evaluations of alisols on inhibiting pancreatic lipase, the IC50 of alisol F 24-acetate (**93**) on pancreatic lipase was 45.5 μM (Cang et al., 2017). Studies results show that alisol B 23-acetate (**47**) can bound plasma protein (Xu et al., 2014).

Alisol A (**1**), alisol A 24-acetate (**2**) and alisol B (**46**) can decrease TG level in plasma by improving lipoprotein lipase (LPL) activity (Xu et al., 2018). The effects of alisols with epoxy aliphatic chain at C-17 on LPL are stronger than those with an open aliohatic chain at C-17. Hydroxyl groups submitted at C-14, C-22, C-28, C-30, and an acetyl group at C-29 are necessary for lipid-regulation action of alisols.

### Anti-inflammatory

Alisol B 23-acetate (**47**) prevents the production of NO in RAW264.7 cells by inhibiting iNOS mRNA expression (Kim et al., 1999). Alisol A 24-acetate (**2**) effectively alleviates liver steatosis by down-regulating SREBP-1c, ACC, FAS genes and up-regulating CPT1 and ACOX1 genes to activate AMPK signaling pathway and inhibit inflammatory cytokines TNF-α, IL-6 levels (Zeng et al., 2016). In addition, alisol B (**46**) and alisol B 23-acetate (**4**7) significantly inhibit the production of leukotriene and the release of β-hexosaminidase in the concentrations of 1–10 mM (Lee et al., 2012).

### Antibacterial

Alisol B (**46**), alisol B 23-acetate (**47**), alisol C 23-acetate (**59**), and alisol A 24-acetate (**2**) have significant bacteriostatic actions on four gram positive and four gram negative antibiotic resistant strains with the MICs ranged from 5 to 10 μg/ml (Jin et al., 2012). In addition, alisol A (**1**), 25-o-ethylalisol A (**16**), 11-deoxyalisol A (**4**), alisol E 24-acetate (**15**) and 25-anhydroalisol F (**97**) fight off gram-positive strains of bacillus subtilis and staphylococcus aureus with MICs ranged from 12.5 to 100 mg/ml (Ma et al., 2016).

### Antiviral

Studies have shown that alisols from A. orientale exhibit obvious anti-hepatitis b virus effect (Jiang et al., 2006). Alisol F (**92**) and alisol F 24-acetate (**93**) significantly inhibit the secretion of HBV surface antigen with an IC_50_ value of 7.7 and 0.6 μM, and HBVe antigen secretion with an IC_50_ value of 5.1 and 8.5 μM, respectively. A series of derivatives of alisol A (**1**) obtained after structural modification also showed potential effect (Zhang et al., 2008, 2009).

## Structure Modification

Alisol B 23-acetate can induce apoptosis and autophagy in cancer cell lines (Xu et al., 2015), and structure modification on alisol B 23-acetate (**47**) allows to obtain a diverse of derivatives (Lee et al., 2002). Alisol B 23-acetate (**47**) reacts with m-chloroperoxybenzoic acid (mCPBA) in CH_2_Cl_2_ at room temperature to gain 13β, 17β-epoxy-23-acetate-alisol B (**67**), and reacts with NH_2_OH.HCl in pyridine and MeOH to achieve amination at C-3. Deacetylation of alisol B 23-acetate (**47**) by NaOH yields alisol B (**46**). Although there is no significant difference of inhibition effect on B16-F10 and HT1080 cell lines between 13β, 17β-epoxy-23-acetate-alisol B (**67**) (ED50 values of 17 and 18 μg/ml) and alisol B 23-acetate (**47**) (ED50 values of 20 μg/ml, respectively), alisol B (**46**) (B16-F10 and HT1080 with ED50 values of 5.2 and 3.1 ug/ml), amination at C-3 of alisol B 23-acetate (**47**) (with ED50 values of 7.5 and 5.1 ug/ml) show exhibited greater activation against B16-F10 and HT1080 cancer cells. It indicates that deacetylation of C-23 and amination at C-3 significantly enhance the inhibition effect on B16-F10 and HT1080 cell lines.

Four hydroxyl groups of alisol A (**1**) are usually the target sites for modification by reacting with acetic anhydride in *N, N*′- dicyclohexylcarbodiimide and 4-dimethylamnopyridine. Alisol A (**1**) can also dehydrate by SOCl_2_ in the presence of anhydrous pyridine. The assessments of anti-hepatitis B virus (HBV) activities suggest alisol A (**1**) analogs with acetoxyl groups at C-11, C-23, C-24 or the epoxy ring at C-13 and C-17 increase the effects on HBV. Dehydration at C-25/C-26 enhances its sensitivity on HBV (Zhang et al., 2008, 2009).

Biotransformation of alisol A (**1**) also derives a series of active compound by several bacteria strains, such as *C. elegans* AS 3.2028 and *P. janthinellum* AS 3.510. Alisol A (**1**) can inhibit the proliferation of HCE-2 cells on the IC_50_ of 99.65 ± 2.81 μM (Zhang et al., 2017). The activity screening results reveal hydroxylation at C-7 and C-12 increases the inhibiting effects of alisol A (**1**) on human carboxylesterase 2 (IC_50_ values of 7.39 ± 1.21 and 3.73 ± 0.76 μM) and the acetyl group at C-23 or C-24 also increases its inhibition effect on HCE-2 cells (IC_50_ values of 3.78 ± 0.21 and 6.11 ± 0.46 μM).

Taken together, epoxidation at C-13 and C-17, hydroxylation at C-23, C-7/C-12, amination at C-3, and dehydration at C-25/C-26 contribute to the activities of protostane tetracyclic skeleton of *A. orientale*, including anticancer activity, anti-hepatitis B virtus, and the inhibiting activity on human carboxylesterase 2.

## Conclusion

The present work systematically summarized the information concerning the phytochemistry, bioactivities and structure modification of triterpenoids in *Alisma* species. To date, more than 100 protostane-type terterpenoids have been isolated and identified. Alisols are reported with anticancer, lipid-regulating, anti-inflammatory, antibacterial, and antiviral activities. Structure modification might contribute to the investigation of the therapeutic potential of alisols.

## Author Contributions

MJ designed the review and was responsible for the study conception. PW and MJ wrote the paper. PW, TS, and RS contributed to summarizing the phytochemistry and structure modification studies on triterpenoids. MH, RW, and JL contributed to summarizing the bioactivity studies on triterpenoids.

## Conflict of Interest

The authors declare that the research was conducted in the absence of any commercial or financial relationships that could be construed as a potential conflict of interest.
